# “What” and “when” predictions modulate auditory processing in a mutually congruent manner

**DOI:** 10.3389/fnins.2023.1180066

**Published:** 2023-09-15

**Authors:** Drew Cappotto, Dan Luo, Hiu Wai Lai, Fei Peng, Lucia Melloni, Jan Wilbert Hendrik Schnupp, Ryszard Auksztulewicz

**Affiliations:** ^1^Department of Neuroscience, City University of Hong Kong, Kowloon, Hong Kong SAR, China; ^2^Ear Institute, University College London, London, United Kingdom; ^3^Department of Neuroscience, Max Planck Institute for Empirical Aesthetics, Frankfurt am Main, Germany; ^4^Department of Neurology, NYU Grossman School of Medicine, New York, NY, United States; ^5^Department of Education and Psychology, Freie Universität Berlin, Berlin, Germany

**Keywords:** auditory neuroscience, predictive coding, temporal processing, electroencephalography, dynamic causal modeling

## Abstract

**Introduction:**

Extracting regularities from ongoing stimulus streams to form predictions is crucial for adaptive behavior. Such regularities exist in terms of the content of the stimuli and their timing, both of which are known to interactively modulate sensory processing. In real-world stimulus streams such as music, regularities can occur at multiple levels, both in terms of contents (e.g., predictions relating to individual notes vs. their more complex groups) and timing (e.g., pertaining to timing between intervals vs. the overall beat of a musical phrase). However, it is unknown whether the brain integrates predictions in a manner that is mutually congruent (e.g., if “beat” timing predictions selectively interact with “what” predictions falling on pulses which define the beat), and whether integrating predictions in different timing conditions relies on dissociable neural correlates.

**Methods:**

To address these questions, our study manipulated “what” and “when” predictions at different levels – (local) interval-defining and (global) beat-defining – within the same stimulus stream, while neural activity was recorded using electroencephalogram (EEG) in participants (*N* = 20) performing a repetition detection task.

**Results:**

Our results reveal that temporal predictions based on beat or interval timing modulated mismatch responses to violations of “what” predictions happening at the predicted time points, and that these modulations were shared between types of temporal predictions in terms of the spatiotemporal distribution of EEG signals. Effective connectivity analysis using dynamic causal modeling showed that the integration of “what” and “when” predictions selectively increased connectivity at relatively late cortical processing stages, between the superior temporal gyrus and the fronto-parietal network.

**Discussion:**

Taken together, these results suggest that the brain integrates different predictions with a high degree of mutual congruence, but in a shared and distributed cortical network. This finding contrasts with recent studies indicating separable mechanisms for beat-based and memory-based predictive processing.

## Introduction

The ability to predict future events based on sensory information is an integral aspect of adaptive sensory processing. Real-world events are complex, consist of statistical regularities, and contain multiple features over which predictions can be formed (Dehaene et al., 2015). In the auditory domain, “what” and “when” predictions are present in virtually every stimulus stream, and their manipulation has been the foundation for numerous studies of predictive coding (Heilbron and Chait, 2018). “What” predictions are typically manipulated by introducing unexpected sensory deviants (oddballs), and comparing the neural responses to the unexpected vs. expected stimuli. In such oddball paradigms, the resulting classical mismatch response (MMR) is interpreted in predictive coding models as resultant error signal (Garrido et al., 2009a,b). As opposed to “what” predictions, which often rely on MMR-based explanations, “when” predictions can be explained by neural entrainment – phase alignment of neural activity to an external temporal structure (Schroeder and Lakatos, 2009; Haegens and Zion Golumbic, 2018; Auksztulewicz et al., 2019) – although this hypothesis is subject to an ongoing debate (Doelling and Assaneo, 2021). However, a fixed rhythmic pulse or beat is not required to form “when” predictions, as multiple temporal cues exist for the formation of such predictions (Morillon et al., 2016; Nobre and van Ede, 2018). Indeed, recent studies have demonstrated that while neural entrainment may be implicated in beat-based predictions, the relative timing between events can be seen as a mnemonic task relying on separable neural mechanisms (Breska and Deouell, 2017; Bouwer et al., 2020).

Several studies have investigated predictions through independent manipulation of timing and content predictability, suggesting interactive and partly dissociable neural correlates and putative underlying mechanisms (Kotz and Schwartze, 2010; Arnal and Giraud, 2012; Buzsaki and Friston, 2016; Auksztulewicz et al., 2018; Chung et al., 2022). In simple sound sequences, MMR amplitudes are typically modulated by “when” predictions, such that deviant-evoked activity is higher when deviants are presented in temporally predictable (e.g., rhythmic/isochronous) sequences (Yabe et al., 1997; Takegata and Morotomi, 1999; Todd et al., 2018; Lumaca et al., 2019; Jalewa et al., 2020). In the auditory domain, such interactions have been suggested to rely on partially dissociable networks mediating “what” and “when” predictions (Hsu et al., 2013), while also jointly modulating stimulus-evoked activity in the superior temporal gyrus (Auksztulewicz et al., 2018). More generally, it has been proposed that interactions between “what” and “when” predictions are inherent to the processing of musical sequences (Musacchia et al., 2014). In this context, it has been suggested that neural entrainment along the non-lemniscal (secondary) auditory pathway (sensitive to the rhythmic sequence structure) can modulate activity in the lemniscal (primary) pathway (encoding stimulus contents), including MMR processing.

However, it is unknown if interactions between “what” predictions (in the lemniscal pathway) and “when” predictions (in the non-lemniscal pathway) depend on different types of predictions present in complex naturalistic stimuli such as speech or music (Hasson et al., 2015). In the case of naturalistic music stimuli (Koelsch et al., 2019), lower-level “what” predictions can be formed about single notes within a sequence, while higher-level predictions can relate to the resulting melody contour, each occurring at their respective time scales. Similarly, “when” predictions can be formed about specific temporal intervals vs. global beat patterns present in melodies. In principle, neural entrainment to a particular time scale might boost the processing of any stimuli presented in the expected time window (Auksztulewicz et al., 2019). Accordingly, it has been shown that beat-based predictions are associated with obligatory resonance-like patterns of neural activity (Breska and Deouell, 2017) and a modulation of stimulus-evoked responses to both relevant and irrelevant stimuli (Breska and Deouell, 2016), while interval-based predictions have been proposed to be more flexible with regards to resource allocation (Breska and Deouell, 2017). However, if entrainment to slower (i.e., more global, beat-based) temporal scales is functionally related to chunking (Ding et al., 2016; Henin et al., 2021), it may show a specific modulation of the processing of stimulus chunks, rather than single elements. Thus, based on current hypotheses of temporal predictions, it is unclear if “when” predictions modulate the processing of stimulus contents (and the respective “what” predictions) in a contextually specific way – e.g. if temporal predictions amplify the processing of any stimuli presented at a preferred time window, or only those stimuli whose contents can be predicted at the corresponding time scale.

Here, we present streams of tones and independently manipulate content-based and time-based characteristics of the stream in two contexts, while recording EEG in healthy volunteers. Temporal predictability was manipulated at faster, interval-based (~4 Hz) and slower, beat-based (~2 Hz) time scales, while acoustic deviants were introduced at lower levels (e.g., interval-defining elements) and higher levels (e.g., beat-defining elements), to evaluate the independent or interactive effect of “what” and “when” predictive processing across different levels. In the analysis, we focused on testing whether “what” and “when” predictions interactively modulate the ERP amplitude evoked by deviants only when they are mutually congruent with respect to the level of manipulation, or if they also have more general effects (e.g., beat-based “when” predictions modulating “what” predictions of single tones vs. longer segments). We also performed source reconstruction and effective connectivity analysis of the observed effects, to test whether different levels of predictions preferentially engage different cortical regions. This integrative approach builds upon previous studies that have separately explored the neural substrates of ‘what’ and ‘when’ predictions, and seeks to advance our understanding of how their underlying mechanisms may dynamically modulate the functional coupling between specific cortical regions.

## Methods

EEG was recorded during an auditory repetition detection task in order to gage (1) the effects of “when” predictions at higher and lower temporal scales on tone-evoked responses and on neural entrainment, as well as (2) the modulatory effect of “when” predictions on the neural signatures of higher and lower-level “what” predictions (MMRs). The use of musical sequences (ascending or descending musical scales) was chosen to reduce the influence of speech-specific processing on neural activity (e.g., modulation by language comprehension, speech-specific semantic and syntactic processing, etc.) and provide a better comparison to similar work in animal models (Jalewa et al., 2020). In the analysis, we focused on interactions between “what” and “when” predictions, specifically testing whether MMRs are modulated by temporal predictability in a contextually specific way (such that beat-based “when” predictions selectively modulate MMRs to violations of “what” predictions falling on beat). To explain the effects observed at the scalp level, we used source reconstruction and model-driven data analysis techniques (dynamic causal modeling), which allowed us to infer the putative mechanisms of interactions between “what” and “when” predictions.

### Participant sample

Participants (*N* = 20, median age 21, range 19–25, 10 females, 10 males; 19 right-handed, 1 left-handed) volunteered to take part in the study at City University of Hong Kong upon written consent. The work was conducted in accordance with protocols approved by the Human Subjects Ethics Sub-Committee. All participants self-reported normal hearing and no current or past neurological or psychiatric disorders.

### Stimulus design and behavioral paradigm

An experimental paradigm was designed in which auditory sequences were manipulated with respect to “what” and “when” predictions at two levels (“what” predictions of interval-defining elements vs. beat-defining elements; interval-based “when” predictions at ~4 Hz vs. beat-based “when” predictions at ~2 Hz), allowing for an analysis of their interactions at each level. To ensure that participants paid attention to stimulus sequences, a decoy task was employed that introduced occasional repetitions, with participants instructed to listen out for such repetitions and respond when they were detected (see below).

Auditory sequences were generated using Psychtoolbox for MATLAB (version 2021a) and delivered to participants fitted with Brainwavz B100 earphones via a TDT RZ6 multiprocessor at a playback sampling rate of 24,414 Hz. Participants were seated in a sound-attenuated EEG booth. Visual stimuli (fixation cross) and instructions were presented on a 24-inch computer monitor and delivered using the Psychophysics Toolbox for MATLAB. Participants were asked to minimize movements and eye blinks and instructed to perform a tone repetition detection task, by pressing a keyboard button using their right index finger as soon as possible upon hearing an immediate tone repetition.

**Figure 1:**
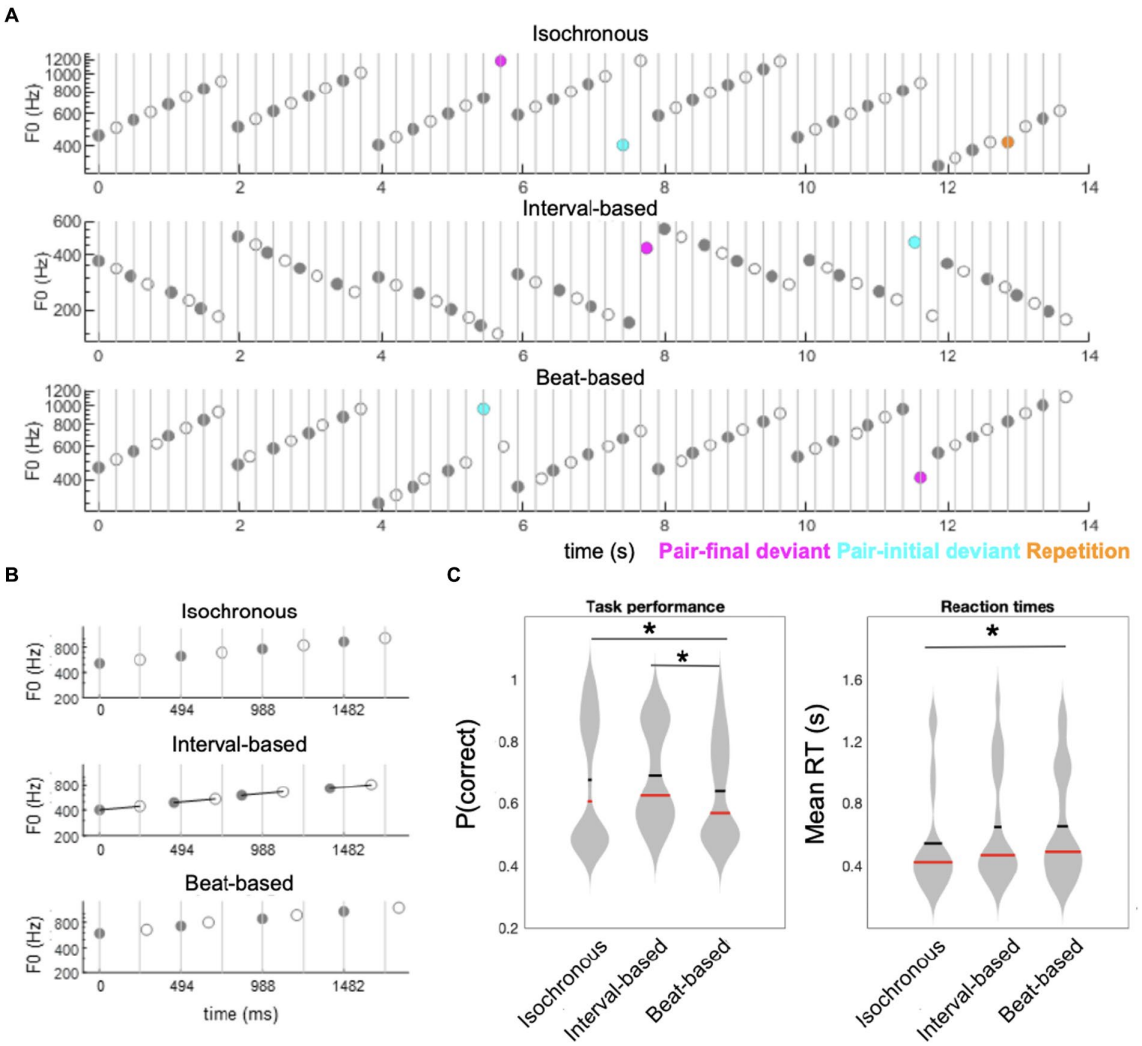
Experimental paradigm and behavioral results. **(A)** Participants listened to sequences of ascending (as represented on the figure) or descending scales of acoustic tones. Sequences were composed of tone pairs, where odd tones (gray circles) were louder than even tones (white circles). Participants performed a tone repetition detection task (orange circles: behavioral targets; presented in a subset of trials). Additionally, sequences could include ID deviants tones (magenta circles), in which one of the pair-final tones had an outlier fundamental frequency (F0), and BD deviants (cyan circles), in which one of the stressed, pair-initial, tones had an outlier F0. **(B)** Sequences were blocked into three temporal conditions: an isochronous condition (upper panel), in which ISI between tones was fixed at 0.247 s; a interval-based condition (middle panel), in which the ISI between odd and even tones within pairs was fixed at 0.247 s but the ISI between odd tones (pair-initial tones) was jittered; and a beat-based condition (lower panel), in which ISI between odd tones (pair-initial tones) was fixed at 0.494 s but the ISI between odd and even tones within pairs was jittered. **(C)** Behavioral results. Left panel: accuracy, right panel: reaction times. Horizontal red bars denote medians across participants; horizontal thick black bars denote means across participants. Asterisks denote *p* < 0.05.

Stimuli were presented in sequences of 7 ascending or descending scales. Each scale was composed of 8 tones equally spaced on a logarithmic scale to form one octave. Thus, across 7 scales a total of 56 tones were presented per sequence (Figures 1A,B). A trial was defined as the presentation of a sequence of 7 scales. Within a trial, all scales were either ascending or descending. The ascending and descending trials were presented in a random order. Each participant heard a total of 240 sequences (trials). The initial tone of each scale was randomly drawn from a frequency range of 300–600 Hz. Each tone was generated by resynthesizing a virtual harp note F4 (played on virtualpiano.net), to match a fixed 166 ms duration and the fundamental frequency used at a given position in the scale. The tone manipulations were implemented in an open-source vocoder, STRAIGHT (Kawahara, 2006) for Matlab 2018b (MathWorks; RRID: SCR_001622). The beat was defined by manipulating the intensity ratio of odd/even tones to form duples, with the even (2nd, 4th, 6th and 8th) tones within a scale presented 10 dB quieter relative to the odd-position tones (Kotz et al., 2018). This amplitude relationship forming the duples was maintained in both control and experimental blocks, as discussed below.

Manipulation of temporal predictability formed three conditions: in the fully-predictable (isochronous) control condition, tones were presented with a fixed ISI (inter-stimulus interval) of 247 ms, resulting in all tones having predictable timing at both the interval-defining time scale (ID) and the beat-defining time scale (BD). In the beat-based (predictable BD, unpredictable ID) condition, the beat/pulse was predictable (corresponding to a fixed pair onset asynchrony, i.e., a fixed 494 ms interval between the onsets of the odd, beat-defining tones) but interval timing was unpredictable (corresponding to a random onset of the even, pair-final tones, relative to the pair-initial tones). In this condition, the exact ISI of the pair-final tones was set by randomly drawing one value from the following 4 ISIs, relative to the standard 247 ms ISI: 33.3% shorter; 16.6% shorter; 16.6% longer; 33.3% longer. Finally, in the interval-based (predictable ID, unpredictable BD) condition, the timing between notes in a duple was predictable (corresponding to a fixed 247 ms ISI of the pair-final tones, relative to the pair-initial tones) but the overall beat/pulse was unpredictable (corresponding to a random onset of the odd, pair-initial tones, relative to the expected 494 ms interval). In this condition, the exact ISI of the pair-initial tones was set by randomly drawing one value from the same 4 ISIs as above, and shifting the onset of the pair-initial tone by this value, relative to the expected 494 ms interval relative to the previous pair onset. A fixed inter-trial interval of 1 s was employed between the offset of the last tone of a 56-tone sequence and the onset of the first tone in the next sequence. The three timing conditions were administered in 12 blocks of 20 trials (4 blocks per condition). Blocks were pseudo-random in order, allowing no immediate repetitions of the same timing condition.

Content predictability was manipulated by altering the fundamental frequency of a subset of tones within the scales. The ID deviants were introduced by replacing the final tone of a scale with an outlier frequency (i.e., a tone whose fundamental frequency was 20% lower/higher than the range of the entire scale). The BD deviants were introduced by replacing the penultimate tone of a scale (i.e., the initial tone of the final pair) in the same manner. Such manipulations allowed us to test for differences in responses evoked by ID deviants (pair-final tone) vs. non-ID deviants (penultimate/pair-initial tone) in interval-based sequences, and BD deviants (penultimate/pair-initial tone) vs. non-BD deviants (pair-final tone) in beat-based sequences.

To facilitate the extraction of statistical regularities in the sequences, in each trial, the first two scales were left unaltered. Two deviant tones were randomly placed within the subsequent 5 scales. Additionally, in 50% of the trials, a scale containing an immediate tone repetition was included in the last 5 scales. In subsequent EEG analysis, neural responses evoked by ID and BD deviants were compared with neural responses evoked by the respective standard tones, designated as the final and penultimate tones in two unaltered scales out of the final 5.

In total, 64.3% of the scales were left unaltered, 14.3% contained an ID deviant, 14.3% contained a BD deviant, and 7.1% contained a tone repetition. The global deviant probability equaled 3.57% of all tones, amounting to 80 deviant tones per deviant type (ID, BD) per temporal condition (isochronous, interval-based, beat-based). To ensure that the EEG analysis was not confounded by differences in baseline duration between temporal conditions (e.g., ID deviants preceded by shorter/longer ISIs in the beat-based condition than in the other two conditions), the ISIs preceding all deviant tones and designated standard tones were replaced by a fixed 247 ms ISI. Therefore, the temporal predictability manipulation was limited to tones surrounding the analyzed tones, and no significant effects were found on the exact timing of either deviants or standards.

Prior to experimental blocks, participants were exposed to a training session consisting of isochronous sequences containing a tone repetition, to familiarize themselves with the task and stimuli. Participants performed training trials until they could detect tone repetition in 3 consecutive trials with reaction times shorter than 2 s. Then, during the actual experiment, participants received feedback on their mean accuracy and reaction time after each block of 20 trials. The data segments (scales) containing tone repetition were subsequently discarded from EEG analysis.

### Behavioral analysis

Analysis was performed on the accuracy and reaction time data corresponding to participant responses during the decoy repetition detection task. Reaction times longer than 2 s were excluded from analysis. Mean reaction times (from correct trials only) were log-transformed to approximate a normal distribution. Accuracy and mean reaction times were entered into separate repeated-measures ANOVAs with a within-subjects factor Time (isochronous, interval-based, beat-based). Post-hoc comparisons were implemented using paired t-tests in MATLAB and corrected over three comparisons for accuracy and three comparisons for reaction times using false discovery rate of 0.05.

### Neural data acquisition and pre-processing

EEG signals were collected using a 64-channel ANT Neuro EEGo Sports amplifier at a sampling rate of 1,024 Hz with no online filters. The recorded data were pre-processed using the SPM12 Toolbox (version 7,219; Wellcome Trust Center for Neuroimaging, University College London; RRID: SCR_007037) for MATLAB (version R2018b). Continuous data were high-pass filtered at 0.1 Hz and notch filtered between 48 Hz and 52 Hz before being down-sampled to 300 Hz and subsequently low-pass filtered at 90 Hz. All filters were 5th order zero-phase Butterworth. Eyeblink artifacts were detected using channel Fpz and removed by subtracting the two top spatiotemporal principal components of eyeblink-evoked responses from all EEG channels (Ille et al., 2002). Cleaned signals were re-referenced to the average of all channels, as is recommended for source reconstruction and dynamic causal modeling (Litvak et al., 2011). The pre-processed data were analyzed separately in the frequency domain (phase coherence analysis) and in the time domain (event-related potentials; ERPs).

### Phase coherence analysis

To test whether tone sequences are associated with dissociable spectral peaks in the neural responses at the element rate (4.048 Hz) and at the pair-rate (2.024 Hz), we analyzed the data in the frequency domain. Continuous data were segmented into epochs ranging from the onset to the offset of each trial (tone sequence). For each participant, channel, and sequence, we calculated the Fourier spectrum of EEG signals measured during that sequence. Based on previous literature, we then calculated the inter-trial phase coherence (ITPC), separately for each temporal condition (isochronous, interval-based, beat-based) according to the following equation (Ding and Simon, 2013) in order to infer phase consistency in each condition:


$$ITPC_{f}=\left({\left[\Sigma^{N}\cos\phi_{f}\right]}^{2}+{\left[\Sigma^{N}\sin\phi_{f}\right]}^{2}\right)/N\;,$$

where *φ_f_* corresponds to the Fourier phase at a given frequency *f,* and *N* corresponds to the number of sequences (80 per condition). We used ITPC also in the aperiodic condition, as it has been shown that consistent slow ramping EEG activity can yield significant ITPC values even in aperiodic (interval-based) sequences (Breska and Ivry, 2020). The same method was used to estimate the stimulus frequency spectrum by calculating the ITPC based on the raw stimulus waveform.

In the initial analysis, ITPC estimates were averaged across EEG channels. To test for the presence of statistically significant spectral peaks, ITPC values at the element-rate (4.048 Hz) and pair-rate (2.024 Hz) were compared against the mean of ITPC values at their respective neighboring frequencies (element-rate: 3.974 and 4.124 Hz; pair-rate: 1.949 and 2.099 Hz) using paired t-tests.

Furthermore, to test whether element-rate and pair-rate spectral peaks observed at single EEG channels show modulations due to temporal predictability, spatial topography maps of single-channel ITPC estimates were converted to 2D images, smoothed with a 5 × 5 mm full-width-at-half-maximum (FWHM) Gaussian kernel (matching the expected spatial scale of EEG scalp data), and entered into repeated-measures ANOVAs (separately for element-rate and pair-rate estimates) with a within-subjects factor Time (isochronous, interval-based, beat-based), implemented in SPM12 as a general linear model (GLM). To account for multiple comparisons and for ITPC correlations across neighboring channels, statistical parametric maps were thresholded at *p* < 0.001 and corrected for multiple comparisons over space at a cluster-level *p*_FWE_ < 0.05 under random field theory assumptions (Kilner et al., 2005). Repeated-measures parametric tests were chosen following previous literature using ITPC (Sokoliuk et al., 2021), under the assumption that differences in ITPC values between conditions are normally distributed. Exact cluster-level *p*-values are only reported for significant clusters, as in statistical parametric mapping the cluster-level p-values depend on uncorrected thresholding and are only computed for the surviving data points. Post-hoc tests were implemented at a Bonferroni-corrected FWE threshold (0.05/3 pairwise comparisons per rate).

Finally, to test whether spectral signatures of temporal predictability are modulated by experience with stimuli, we split the data into two halves (two consecutive bins of 40 trials), separately for each condition. Element-rate and pair-rate ITPC estimates were averaged across EEG channels and compared separately for each of the two halves using repeated-measures ANOVAs with a within-subjects factor Time (isochronous, interval-based, beat-based). Post-hoc comparisons were implemented using paired t-tests and corrected over 4 comparisons (element/pair-rate; first/s half) using false discovery rate of 0.05.

### Event-related potentials

For the time-domain analysis, data were segmented into epochs ranging from-50 ms before to 247 ms after deviant/standard tone onset, baseline-corrected from-25 ms to 25 ms to prevent epoch contamination due to the temporally structured presentation (Fitzgerald et al., 2021), and denoised using the “Dynamic Separation of Sources” (DSS) algorithm (de Cheveigné and Simon, 2008), reducing the influence of noisy channels. Condition-specific ERPs (corresponding to ID/BD deviants and the respective standards, presented in each of the three temporal conditions) were calculated using robust averaging across trials (reducing the influence of outlier trials), as implemented in the SPM12 toolbox, and low-pass filtered at 48 Hz (5th order zero-phase Butterworth). The resulting ERPs were analyzed univariately to gage the effects of “what” and “when” predictions on evoked responses. ERP data were converted to 3D images (2D: spatial topography; 1D: time), and the resulting images were spatially smoothed using a 5 × 5 mm FWHM Gaussian kernel. The smoothed images were entered into a general linear model (GLM) implementing a 3 × 3 repeated-measures ANOVA with a within-subject factors Contents (standard, deviant ID, deviant BD) and Time (isochronous, interval-based, beat-based). Parametric tests based on a GLM are an established method of analyzing ERP data (Litvak et al., 2011). Beyond testing for the two main effects and a general 3 × 3 interaction, we also designed a planned contrast quantifying the congruence effect (i.e., whether “when” predictions specifically modulate the amplitude of mismatch signals evoked by deviants presented at a time scale congruent with “when” predictions – specifically, deviant ID in the interval-based condition and deviant BD in the beat-based conditions). To this end, we tested for a 2 × 2 interaction between Contents (deviant ID, deviant BD) and Time (interval-based, beat-based). To account for multiple comparisons as well as for ERP amplitude correlations across neighboring channels and time points, statistical parametric maps were thresholded at *p* < 0.001 and corrected for multiple comparisons over space and time at a cluster-level *p*_FWE_ < 0.05 under random field theory assumptions (Kilner et al., 2005). Post-hoc tests were implemented at a Bonferroni-corrected FWE threshold (0.05/3 pairwise comparisons).

### Brain-behavior correlations

**Figure 2:**
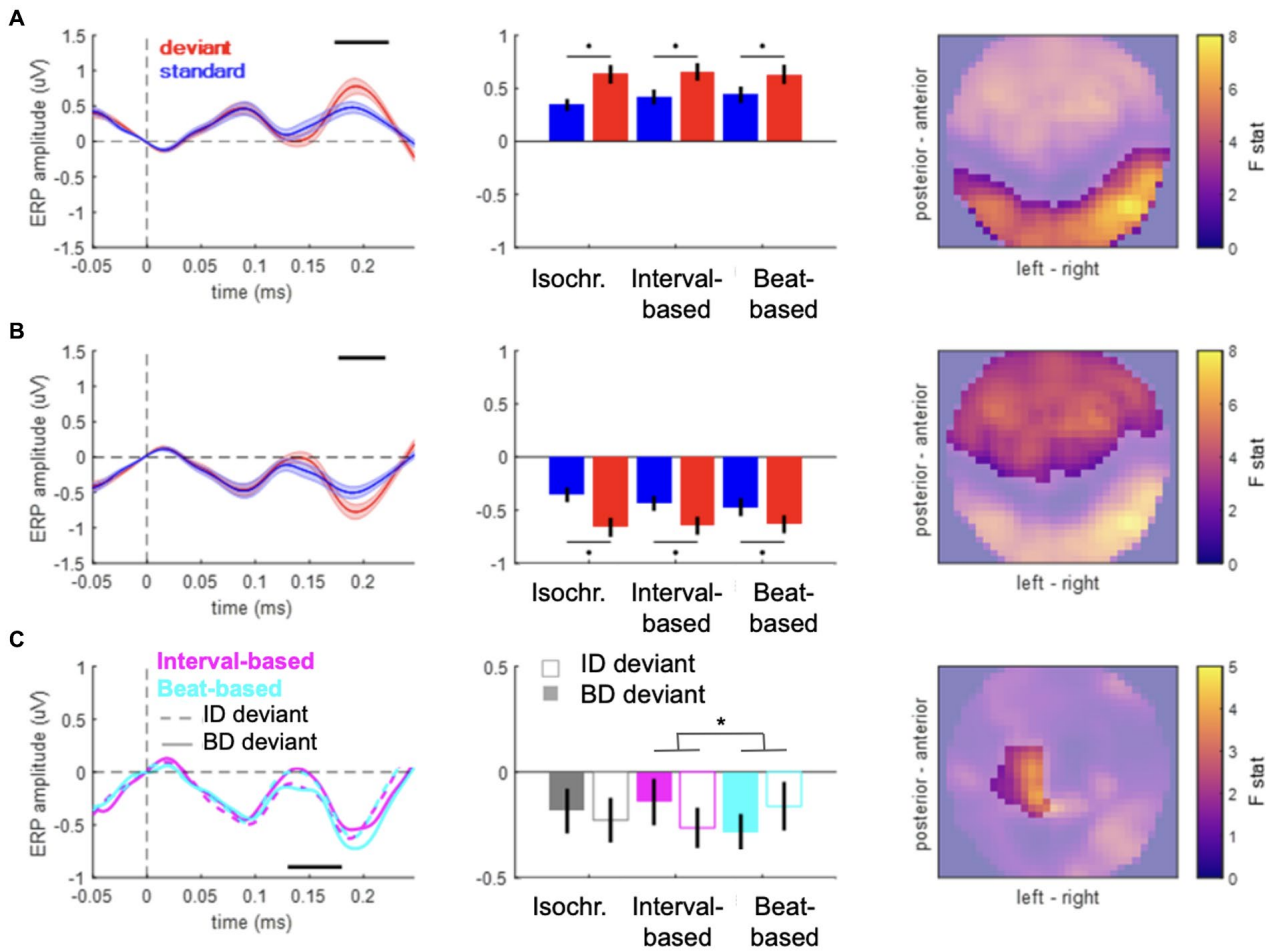
Event-related potentials. **(A,B)** Main effect of content-based predictions (deviant vs. standard) in anterior **(A)** and posterior **(B)** clusters. Left panels: time courses of ERPs averaged over the spatial topography clusters shown in the right panels. Shaded area denotes SEM across participants. Black horizontal bar denotes *p*_FWE_ < 0.05. Middle panels: mean voltage values for standards (blue) and deviants (red). Right panels: spatial distribution of the main effect. Color bar: F value. **(C)** Contextual interaction between content-based predictions (deviant ID vs. deviant BD) and temporal predictions (beat-based vs. interval-based). Left panels: time courses of ERPs averaged over the spatial topography clusters shown in the right panels. Black horizontal bar denotes *p*_FWE_ < 0.05. Middle panels: mean voltage values for the six deviant conditions. Right panels: spatial distribution of the interaction effect. Color bar: F value.

**Figure 3:**
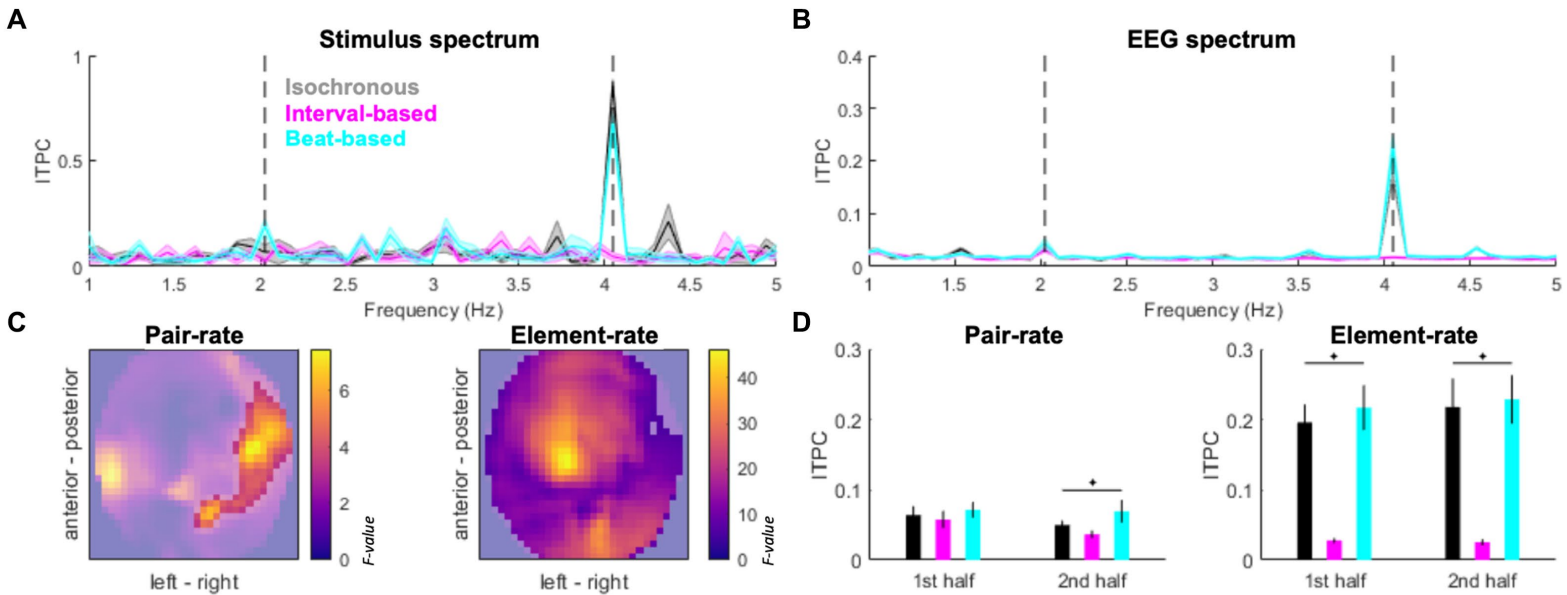
Spectral signatures of temporal predictability. **(A)** Inter-trial phase coherence (ITPC) in the stimulus spectrum. Black: isochronous, cyan: beat-based, magenta: interval-based. Pair-rate (2.024 Hz) and element-rate (4.048 Hz) peaks are indicated by dashed vertical lines. **(B)** ITPC based on EEG activity (averaged across channels). Legend as in Panel A. Shaded areas indicate SEM across participants. **(C)** EEG topography maps of main effects of Condition (isochronous vs. interval-based vs. beat-based) on the pair-rate peak ITPC (left panel) and element-rate peak ITPC (right panel). Statistical F values are represented on the color scale. Unmasked area corresponds to significant clusters (*p*_FWE_ < 0.05). **(D)** Pair-rate (left panel) and element-rate (right panel) peak ITPC values plotted separately for the 1st half and 2nd half of the trials. Error bars denote SEM across participants.

To test whether the neural effects of “what” and/or “when” predictive processing correlate with each other, as well as with behavioral benefits of “when” predictions in the repetition detection task, we performed a correlation analysis across participants. Thus, for each participant, we calculated a single behavioral index (the difference between accuracy scores obtained in the interval-based vs. beat-based condition; chosen given a significant difference between these two conditions, see Results) and three statistically significant neural indices. The second neural index – the “ITPC effect” – quantified the difference between the pair-rate ITPC values obtained for interval-based vs. beat-based conditions in the second half of the experiment (where a significant difference was found; see Results Figure 3D). The second neural index – the “congruence effect” – quantified the difference between deviant-evoked ERP amplitudes measured in the temporally congruent condition (deviant ID presented in interval-based; deviant BD presented in beat-based trials) and incongruent conditions (deviant BD presented in interval-based; deviant ID presented in the beat-based trials), averaged across electrodes in the significant cluster where we observed a significant congruence effect (i.e., a 2 × 2 interaction between “what” and “when” predictions; see Results and Figure 2C). The third neural index – the “mismatch effect” – quantified the difference between the absolute deviant-evoked and standard-evoked ERP amplitudes (averaged across significant channels and temporal conditions; Figures 2A,B), since we hypothesized that performance in the repetition detection task might be related to overall deviance detection, we also included an index of “what” predictions. We then fitted a linear regression model with three predictors (i.e., the three neural indices) regressed against the behavioral accuracy index, and identified outlier participants using a threshold of Cook’s distance exceeding 5 times the mean. Correlations between all measures were quantified using Pearson’s *r* and corrected for multiple comparisons using Bonferroni correction, implementing a conservative correction given no *a priori* assumptions about the correlation coefficients.

### Source reconstruction

Source reconstruction was performed under group constraints (Litvak and Friston, 2008) which allows for an estimation of source activity at a single-participant level under the assumption that activity is reconstructed in the same subset of sources for each participant (i.e., reducing the influence of outliers). Sources were estimated using an empirical Bayesian beamformer (Wipf and Nagarajan, 2009; Belardinelli et al., 2012; Little et al., 2018) based on the entire post-stimulus time window (0–247 ms). Since in the ERP analysis (see Results) we identified two principal findings – namely a difference between ERPs evoked by deviants and standards, and an interaction between deviant type and temporal condition – we focused on comparing source estimates corresponding to these effects. In the analysis of the difference between deviants and standards, source estimates were extracted for the 173–223 ms time window, converted into 3D images consisting of 3 spatial dimensions and smoothed with a 10 × 10 × 10 mm FWHM Gaussian kernel (Guitart-Masip et al., 2013). Smoothed images were then entered into a GLM implementing a 3 × 3 repeated-measures ANOVA with within-subjects factors of Content (standard, deviant ID, deviant BD) and Time (isochronous, interval-based, beat-based). Parametric tests based on a GLM are an established method of analyzing EEG source reconstruction maps (Litvak et al., 2011). In the analysis of the interaction between deviant type and temporal condition, source estimates were extracted for the 130–180 ms and processed as above. Smoothed images were then entered into a GLM implementing a 2 × 2 repeated-measures ANOVA with within-subjects factors of Content (deviant ID, deviant BD) and Time (interval-based, beat-based). To account for multiple comparisons as well as for source estimate correlations across neighboring voxels, statistical parametric maps were thresholded and corrected for multiple comparisons over space at a cluster-level *p*_FWE_ < 0.05 under random field theory assumptions (Kilner et al., 2005). Source labels were assigned using the Neuromorphometrics probabilistic atlas, as implemented in SPM12.

### Dynamic causal modeling

Dynamic causal modeling (DCM) was used to estimate source-level connectivity parameters associated with general mismatch processing (deviant vs. standard) and with the contextual interaction between “what” and “when” predictions (deviant ID presented in the interval-based condition, and deviant BD presented in the beat-based condition, vs. deviant ID presented in the beat-based condition, and deviant BD presented in the interval-based condition). DCM is a type of an effective connectivity analysis based on a generative model, which maps the data measured at the sensor level (here: EEG channels) to source-level activity. The generative model comprises a number of sources which represent distinct cortical regions, forming a sparse interconnected network. Activity in each source is explained by a set of neural populations, based on a canonical microcircuit (Bastos et al., 2012), and modeled using coupled differential equations that describe the changes in postsynaptic voltage and current in each population. Here, we used a microcircuit consisting of four populations (superficial and deep pyramidal cells, spiny stellate cells, and inhibitory interneurons), each having a distinct connectivity profile of ascending, descending, and lateral extrinsic connectivity (linking different sources) and intrinsic connectivity (linking different populations within each source). The exact form of the canonical microcircuit and the connectivity profile was identical as in previous literature on the topic (Auksztulewicz and Friston, 2015; Auksztulewicz et al., 2018; Rosch et al., 2019; Fitzgerald et al., 2021; Todorovic and Auksztulewicz, 2021). Importantly for our study, a subset of intrinsic connections corresponds to self-connectivity parameters, describing the neural gain of each region. Both extrinsic connectivity and gain parameters were allowed to undergo condition-specific changes, modeling differences between experimental conditions (deviants vs. standards, and the hierarchical interaction between “what” and “when” predictions). All ascending and lateral connections are modeled to be excitatory, while all intrinsic and descending connections are modeled to be inhibitory.

Here, we used DCM to reproduce the single-participant, condition-specific ERPs in the 0–247 ms range. Based on the source reconstruction (see Results) and previous literature (Garrido et al., 2009a,b), we included six sources in the cortical network: bilateral primary auditory cortex (A1; Montreal Neurological Institute coordinates: left, [−42–22 7]; right, [46–14 8]), bilateral superior temporal gyrus (STG; left, [−60–20 -8]; right, [59–25 8]), right inferior frontal gyrus (IFG; [40 26–6]), and left superior parietal lobule (SPL; [−26–40 46]). To quantify model fits, we used the free-energy approximation to model evidence, penalized by model complexity. The analysis was conducted in a hierarchical manner-first, model parameters (including extrinsic and intrinsic connections, as well as their condition-specific changes) were optimized at the single participants’ level, and then the significant parameters were inferred at the group level.

At the first level, models were fitted to single participants’ ERP data over two factors: “what” predictions (all deviants vs. standards) and the contextual interaction between “what” and “when” predictions (deviant ID presented in the interval-based condition, and deviant BD presented in the beat-based condition, vs. deviant ID presented in the beat-based condition, and deviant BD presented in the interval-based condition). At this level, all extrinsic and intrinsic connections were allowed to be modulated by both factors, corresponding to a “full” model.

Since model inversion in DCM is susceptible to local maxima due to the inherently nonlinear nature of the models, the analysis at the second (group) level implemented parametric empirical Bayes (Friston et al., 2015). Therefore, group-level effects were inferred by (re) fitting the same “full” models to single participants’ data, under the assumption that model parameters should be normally distributed in the participant sample, and updating the posterior distribution of the parameter estimates. This effectively reduces the influence of outlier participants. We used Bayesian model reduction (Friston and Penny, 2011) to compare the “full” models against a range of “reduced” models, in which some parameters were not permitted to be modulated by the experimental factors. Specifically, we designed a space of alternative models, such that each model allowed for a different subset of connections to contribute to the observed ERPs. The model space examined each combination of modulations of (1) ascending connections (e.g., from A1 to STG), (2) descending connections (e.g., from STG to A1), (3) lateral connections (e.g., from left to right STG), and (4) intrinsic connections (i.e., gain parameters). This resulted in 256 models (16 models for each of the two factors). The free-energy approximation to log-model evidence was used to score each model. Since no single winning model was selected (see Results), Bayesian model averaging was used to obtain weighted averages of posterior parameter estimates, weighted by the log-evidence of each model. This procedure yielded Bayesian confidence intervals for each parameter, quantifying the uncertainty of parameter estimates. Parameters with 99.9% confidence intervals falling either side of zero (corresponding to *p* < 0.001) were selected as statistically significant.

## Results

### Behavioral results

Performance across all trials revealed significant differences in accuracy across conditions (main effect of Time: F_2,38_ = 7.3530, *p* = 0.002), corresponding to significantly lower accuracy in the beat-based condition (mean ± SEM: 63.88% ± 3.65%) than in the isochronous (mean ± SEM: 67.75% ± 4.64%; t_19_ = −2.5272, *p* = 0.0205, FDR-corrected) and interval-based conditions (mean ± SEM: 69.12% ± 3.55%; t_19_ = −5.984, *p* < 0.001, FDR-corrected) (Figure 1C). Reaction times also significantly differed across conditions (F_2,38_ = 3.5543, *p* = 0.0385), with post-hoc analysis revealing that reaction times were significantly faster in the isochronous condition (mean ± SEM: 511 ± 74 ms) than in the beat-based condition (mean ± SEM: 653 ± 79 ms; t_19_ = 2.4089, *p* = 0.0263, uncorrected; not significant after FDR correction). The difference between the isochronous condition and the interval-based condition (mean ± SEM: 649 ± 83 ms) showed a nominal but not significant difference (t_19_ = 2.0132, *p* = 0.0585, uncorrected). No significant difference was observed between the beat-based condition and the interval-based condition (*p* = 0.9013).

### Phase coherence analysis

The stimulus spectrum of inter-trial phase coherence (ITPC) differed between experimental conditions, such that (1) a prominent 4.048 Hz peak was found for isochronous sequences; (2) a prominent but relatively weaker 4.048 Hz was accompanied by a minor 2.024 Hz peak for beat-based sequences; (3) no evident peaks were found for the interval-based sequences (Figure 3A). In the EEG spectrum of ITPC (averaged across conditions and channels; Figure 3B), both element-rate peak (4.048 Hz) and pair-rate peak (2.024 Hz) were observed, relative to neighboring frequency points (element-rate: t_19_ = 6.8489, *p* < 0.001; pair-rate: t_19_ = 3.6274, *p* = 0.0018). The element-rate ITPC estimates were higher in the isochronous and beat-based conditions than in the interval-based conditions, and this effect was observed at most of the EEG channels (F_max_ = 46.30, Z_max_ = 6.43, *p*_FWE_ < 0.001; pairwise comparisons: isochronous vs. interval-based, T_max_ = 8.02, Z_max_ = 6.10, *p*_FWE_ < 0.001; beat-based vs. interval-based, T_max_ = 9.62, Z_max_ = 6.81, p_FWE_ < 0.001; isochronous vs. beat-based, all *p*_FWE_ > 0.05; Figure 3C all pairwise tests compared across multiple comparisons by adjusting FWE threshold to 0.05/3). On the other hand, the pair-rate ITPC estimates were higher in the beat-based condition than in the other two conditions, and this effect was observed over right lateral channels (F_max_ = 7.45, Z_max_ = 2.90, *p*_FWE_ = 0.031; pairwise comparisons: beat-based vs. isochronous, T_max_ = 3.81, Z_max_ = 3.48, *p*_FWE_ = 0.004; beat-based vs. interval-based, T_max_ = 3.83, Z_max_ = 3.50, *p*_FWE_ = 0.001; isochronous vs. interval-based, all *p*_FWE_ > 0.05; all pairwise tests compared across multiple comparisons by adjusting FWE threshold to 0.05/3). Interestingly, the pair-rate differences between conditions built up during the experiment: they were not significant during the first half of the experiment (F_2,59_ = 1.0433, *p* = 0.3622), and were only observed during the second half of the experiment (F_2,59_ = 3.8798, *p* = 0.0293, FDR-corrected). This was not the case for the element-rate differences between conditions, which were stable during the experiment (first half: F_2,59_ = 26.1701, *p* < 0.001; second half: F_2,59_ = 26.9480, *p* < 0.001; both FDR-corrected).

Since the pair-rate ITPC estimates showed a laterality-sensitive result, and our participant sample included one left-handed participant, we repeated this analysis after flipping this participant’s ITPC topography maps. This control analysis yielded a virtually identical pattern of results, showing a right-lateralized cluster (F_max_ = 6.59, Z_max_ = 2.70, *p*_FWE_ = 0.048). Therefore, we have decided to include the original topography maps (without flipping) for the left-handed participant in all subsequent analyzes.

The emergence of pair-rate differences in ITPC over the course of the experiment was reflected in behavior. Specifically, RTs decreased for the second half of the experiment, relative to the first half, only for the beat-based condition (Wilcoxon’s signed rank test: Z_19_ = −2.0926, *p* = 0.0364) but not for the isochronous condition (Z_19_ = −1.6902, *p* = 0.0910) or the interval-based condition (Z_19_ = −0.8213, *p* = 0.4115). No differences in accuracy were observed for any of the three conditions across the first and second halves of the experiment (beat-based: Z_19_ = 1.8254, *p* = 0.0679; isochronous: Z_19_ = −0.8551, *p* = 0.3925; interval-based: Z_19_ = 0.6955, *p* = 0.4867).

### Event-related potentials

To test for effects of “what” and “when” predictions on ERP amplitudes, we analyzed the data in the time domain. ERP amplitudes differed significantly between deviant and standard tones, pooled over temporal conditions (Figure 2A, posterior cluster: 173–223 ms, F_max_ = 53.94, Z_max_ = 6.68, *p*_FWE_ < 0.001; Figure 2B, anterior cluster: 177–220 ms; F_max_ = 37.57; Z_max_ = 5.67; *p*_FWE_ < 0.001), corresponding to a typical anterior–posterior MMN topography after common-average referencing (Mahajan et al., 2017). When analyzing specific deviant types (ID and BD deviants vs. their respective standards), significant differences between deviants and standards were observed in both cases (deviants ID vs. standards: posterior cluster, 173–223 ms, F_max_ = 41.50, Z_max_ = 5.94, *p*_FWE_ < 0.001; anterior cluster, 177–227 ms; F_max_ = 35.56; Z_max_ = 5.52; *p*_FWE_ < 0.001; deviants BD vs. standards: posterior cluster, 170–220 ms, F_max_ = 45.63, Z_max_ = 6.20, *p*_FWE_ < 0.001; anterior cluster, 177–213 ms; F_max_ = 30.17; Z_max_ = 5.11; *p*_FWE_ < 0.001). No significant differences were observed between the two deviant types, pooling over temporal conditions (*p*_FWE_ > 0.05). Thus, the main effect of “what” predictions differentiated between deviants and standards, but not between deviant types.

In the analysis of the main effect of “when” predictions (pooled over deviants and standards), no significant differences between the three temporal conditions were revealed (all *p*_FWE_ > 0.05). Similarly, in the analysis of the interaction effect of “what” and “when” predictions (pooled over deviant types), no significant effects were revealed. Specifically, neither deviants nor standards showed significant ERP amplitude differences when presented in different temporal contexts (all *p*_FWE_ > 0.05). Thus, no significant effects were found when testing for the effects of the overall temporal structure of the sound sequences on the element-evoked responses (averaged across deviants and standards) or the mismatch responses (differences between deviants and standards).

However, an analysis of the interaction between “what” and “when” predictions based on deviants presented in congruent temporal contexts (e.g., deviant ID in the interval-based condition) and those presented in non-temporally congruent contexts (e.g., deviant ID in the beat-based condition) revealed a significant interaction between deviant type and temporal condition (Figure 2B; left central-posterior cluster: 130–180 ms, F_max_ = 20.63, Z_max_ = 4.24, *p*_FWE_ = 0.044). Post-hoc analysis revealed that MMR amplitudes in interval-based conditions were significantly larger for deviant ID (mean ± SEM: −0.1640 ± 0.0942 μV) than for deviant BD (mean ± SEM: 0.0091 ± 0.1010 μV; t_19_ = 2.2843, *p* = 0.0340, two-tailed, uncorrected), although this pairwise difference did not survive correction for multiple comparisons across two tests. In the beat-based condition, MMR amplitude was observed to be nominally larger for deviant BD (mean ± SEM: −0.1725 ± 0.0851 μV) than for deviant ID (mean ± SEM: −0.0155 ± 0.1233 μV), although the effect did not reach significance (*t*_19_ = 1.9024, *p* = 0.0724, two-tailed, uncorrected). No significant interaction effects were revealed when comparing deviant types between the isochronous condition and either the beat-based or the interval-based conditions. Thus, we observed an increase in deviant ERP amplitude when deviants were presented in a temporally congruent context, although pairwise differences split between the two temporal conditions did not yield robust effects.

In a control analysis, to ensure that the observed effects are specific to deviant tones, we analyzed the interaction for standard tones (rather than deviant tones). This analysis revealed no significant clusters (all *p*_FWE_ > 0.05 at the cluster level), suggesting that for standard tones, stimulus properties such as loudness and sequence position *per se* do not interact with temporal expectations, and thus the observed congruency effect is specific to deviant stimuli.

### Brain-behavior correlation analysis

Three neural predictors – the “congruence index” (quantifying the interactive effects of “what” and “when” predictions on ERPs), the “ITPC index” (quantifying the effect of “when” predictions on ITPC), and the “mismatch index” (quantifying the effect of “what” predictions on ERPs) – were tested as potential correlates of the behavioral benefits in the repetition detection task accuracy. We identified two outlier participants based on a linear regression model. Having excluded these two participants, we did not find any significant correlations between the neural indices and the behavioral index (Pearson’s *r;* all *p* > 0.2). However, we did find a significant correlation between the congruence index and the ITPC index (*r* = 0.6439; *p* = 0.0039; corrected), such that the magnitude of the ERP difference between deviants presented in the temporally congruent vs. incongruent conditions positively correlated with the magnitude of the ITPC difference between interval-based and beat-based conditions.

### Source reconstruction

**Figure 4:**
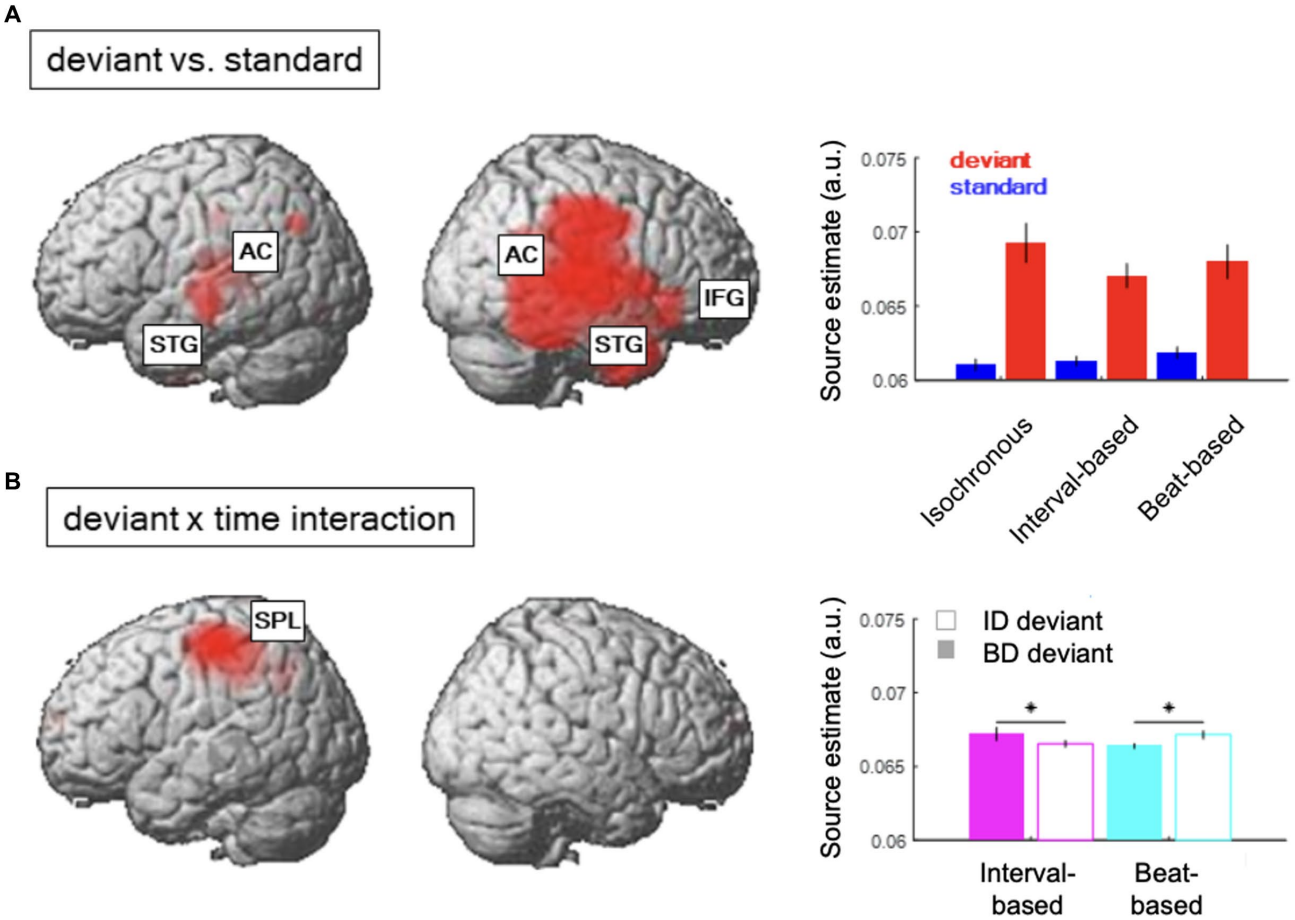
Source reconstruction. **(A)** Regions showing a significant main effect of content-based predictions (deviant vs. standard). Inset shows average source estimates per condition. Error bars denote SEM across participants. **(B)** Regions showing a significant contextual interaction effect between content-based predictions (deviant ID vs. deviant BD) and temporal predictions (interval-based vs. beat-based). Figure legend as in **(A)**.

To identify the most plausible sources underlying the observed ERP differences between deviants and standards, as well as the contextual interaction between deviant types and temporal conditions, we carried out a source reconstruction analysis (Figure 4). Overall, source reconstruction explained 76.43 ± 3.08% (mean ± SEM across participants) of sensor-level variance, consistent with previous literature (Abreu et al., 2022).

The difference between source estimates associated with deviants and standards was localized to a large network of regions (Figure 4A; see Table 1 for full results), including bilateral auditory cortex (AC) and superior temporal gyri (STG) and the right inferior frontal gyrus (IFG). On the other hand, the interaction effect between deviant types and temporal conditions (Figure 4B) was localized to a spatially confined cluster in the left superior parietal lobule (SPL; see Table 1). A post-hoc analysis revealed that, in this cluster, deviant ID responses presented in the interval-based condition were associated with weaker source estimates than deviant BD responses presented in the interval-based condition (T_max_ = 3.67, Z_max_ = 3.46, *p*_FWE_ = 0.009, small-volume corrected). Similarly, deviant BD responses presented in the beat-based condition were associated with weaker source estimates than element deviant responses presented in the beat-based condition (T_max_ = 5.79, Z_max_ = 5.11, *p*_FWE_ = 0.003, small-volume corrected). Thus, while the deviant processing could be linked to a wide network of auditory and frontal regions, deviants presented in the corresponding temporal predictability conditions (e.g., element deviants in the interval-based context) were associated with a relative decrease of left parietal activity.

**Table 1:** Source reconstruction results.

Effect	Cluster label	Peak MNI coords	F_max_	Z_max_	Voxel extent	p_FWE_
Deviant vs. standard	Right transverse temporal gyrus/auditory cortex (AC)	48 -20 12	53.99	4.83	20,508	< 0.001
	Right superior temporal gyrus (STG)	44 -48 12	40.15	4.42		
	Right inferior frontal gyrus (IFG)	40 26 -6	34.52	4.20		
	Left transverse temporal gyrus/auditory cortex (AC)	-38 -28 12	34.31	4.20	2,177	0.003
	Left superior temporal gyrus (STG)	-60 -20 -8	31.19	4.06		
(“ID” vs. “BD” deviant) x (interval-based vs. beat-based)	Left superior parietal lobule (lSPL)	-26 -40 46	49.37	5.82	3,073	0.003

Summary of significant clusters showing differences between conditions.

### Dynamic causal modeling

To infer the most likely effective connectivity patterns underlying the observed ERP results, we used the six main cortical regions identified in the source reconstruction results as regions of interest (ROIs) to build a generative model of the ERP data. A fully interconnected model, fitted to each participants’ ERP data, explained on average 71.03% of the ERP variance (SEM across participants 2.81%), comparable to previous literature (Garrido et al., 2009a,b; Adams et al., 2022).

Bayesian model reduction was used to obtain connectivity and gain parameters of a range of reduced models, in which only a subset of parameters were allowed to be modulated by the two conditions (deviant vs. standard; interaction deviant ID/BD x interval-based/beat-based). Using this procedure, we did not identify a single winning model (difference between the free-energy approximation to log-model evidence between the nominally winning model and the next-best model – i.e., log Bayes factor – was 0.8029, corresponding to model probability of 41.79%). Therefore, we implemented Bayesian model average to integrate model parameter estimates from the entire model space, taking into account the uncertainty about the winning model.

**Figure 5:**
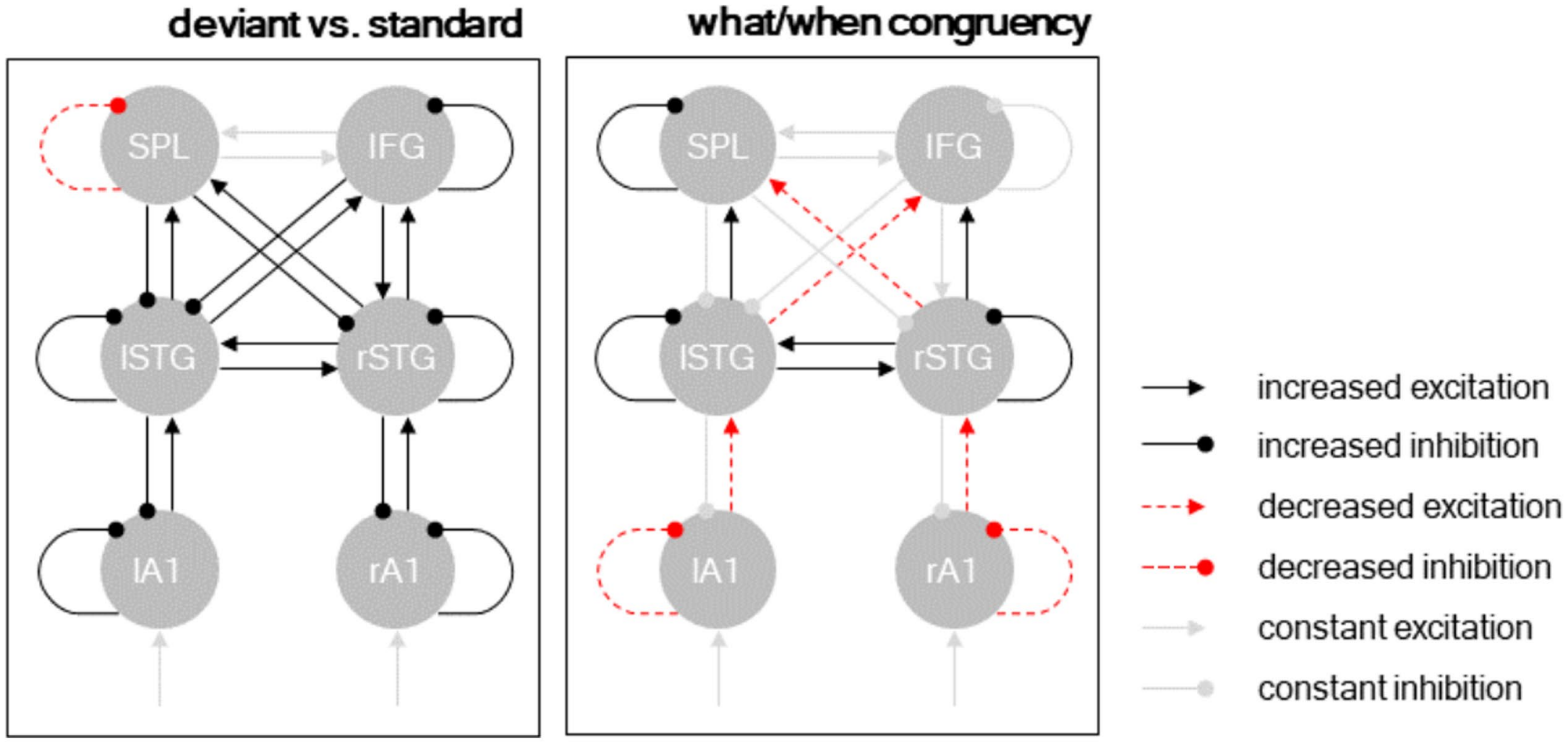
Dynamic causal modeling. Posterior model parameters. Separate panels show different condition-specific effects. Black arrows: excitatory connections; red arrows: inhibitory connections; solid lines: condition-specific increase; dashed lines: condition-specific decrease. Significant parameters (*p* < 0.001) shown in black/red, remaining connections (constant excitation/inhibition) shown in gray.

The posterior parameter estimates of the Bayesian model average are plotted in Figure 5 and reported in Table 2. The results revealed that deviant processing (as opposed to standard processing) significantly increased nearly all connectivity estimates (probability of increase >99.9% for all parameters), corresponding to an increase in excitatory ascending connectivity and in inhibitory descending and intrinsic (gain) connectivity – with the exception of the intrinsic self-inhibition in the left SPL region, which was significantly decreased following deviant processing, as well as the bidirectional connectivity between the left SPL and right IFG, which was not affected by deviant processing.

**Table 2:** Dynamic causal modeling results.

Connection type	Connection label	Effect of content-based predictions (deviant vs. standard) [log mean; log variance]	Effect of contextual interaction (congruent vs. incongruent) [log mean; log variance]
Intrinsic (gain)	lA1- > lA1	50% self-inhibition increase [0.4106; 0.002]	26% self-inhibition decrease [−0.2996; 0.0013]
	rA1- > rA1	9% self-inhibition increase [0.0873; 0.0019]	13% self-inhibition decrease [−0.1360; 0.0013]
	lSTG- > lSTG	44% self-inhibition increase [0.3726; 0.0086]	9% self-inhibition increase [0.0851; 0.0040]
	rSTG- > rSTG	35% self-inhibition increase [0.2950; 0.0095]	33% self-inhibition increase [0.2878; 0.0050]
	lSPL- > lSPL	42% self-inhibition decrease [−0.5492; 0.0259]	12% self-inhibition increase [0.1196; 0.0227]
	rIFG- > rIFG	11% self-inhibition increase [0.1054; 0.0203]	n.s. [−0.0358; 0.0172]
Extrinsic (ascending)	lA1- > lSTG	21% excitation increase [0.1910; 0.0011]	16% excitation decrease [−0.1694; 0.001]
	rA1- > rSTG	13% excitation increase [0.123; 0.001]	10% excitation decrease [−0.1021; 0.001]
	lSTG- > lSPL	33% excitation increase [0.2884; 0.0082]	20% excitation increase [0.1855; 0.0028]
	rSTG- > rIFG	34% excitation increase [0.2971; 0.0074]	33% excitation increase [0.2820; 0.0032]
	lSTG- > rIFG	41% excitation increase [0.3524; 0.0079]	13% excitation decrease [−0.1451; 0.004]
	rSTG- > lSPL	34% excitation increase [0.2960; 0.007]	10% excitation decrease [−0.1098; 0.0029]
Extrinsic (descending)	lSTG- > lA1	140% inhibition increase [0.8746; 0.0136]	n.s. [−0.0001; <0.001]
	rSTG- > rA1	151% inhibition increase [0.9183; 0.013]	n.s. [<0.001; <0.001]
	lSPL- > lSTG	111% inhibition increase [0.7471; 0.009]	n.s. [<0.001; <0.001]
	rIFG- > rSTG	10% inhibition increase [0.0929; 0.008]	n.s. [<0.001; <0.001]
	rIFG- > lSTG	24% inhibition increase [0.2234; 0.0116]	n.s. [<0.001; <0.001]
	lSPL- > rSTG	166% inhibition increase [0.9732; 0.0086]	n.s. [<0.001; <0.001]
Extrinsic (lateral)	lSTG- > rSTG	88% excitation increase [0.6278; 0.0182]	60% excitation increase [0.4618; 0.013]
	rSTG- > lSTG	13% excitation increase [0.1176; 0.029]	20% excitation increase [0.1899; 0.0208]
	lSPL- > rIFG	n.s. [−0.1022; 0.0411]	n.s. [0.0559; 0.0299]
	rIFG- > lSPL	n.s. [−0.0607; 0.0306]	n.s. [−0.028; 0.0225]

Summary of significant condition-specific effects on connectivity estimates. Log mean and variance estimates are reported for completeness. To convert between log means and the more intuitive percentage increase/decrease, log means should be exponentiated and compared to a baseline of 1: e.g., exp (0.4106) = 1.5077 = ca. 51% increase in connection weight.

The interaction between deviant type (deviant ID vs. deviant BD) and temporal predictability (beat-based vs. interval-based) modulated a more nuanced connectivity pattern. At the hierarchically lower level (between A1 and STG), deviants processed in a temporally congruent condition (i.e., deviant ID in the interval-based condition, and deviant BD in the beat-based condition) decreased excitatory ascending connectivity from A1 to STG and inhibitory self-connectivity in A1. Conversely, at the hierarchically higher level (between STG and the fronto-parietal regions), deviants processed in a temporally congruent condition increased excitatory ascending connectivity from STG to SPL/IFC and inhibitory self-connectivity in the STG. Furthermore, deviants processed in a temporally congruent condition (1) increased lateral connectivity between the left and right STG, (2) decreased cross-hemispheric ascending connectivity between the STG regions and the fronto-parietal regions, and (3) increased self-inhibition in the left SPL region.

## Discussion

In the present study, we found that “when” predictions modulate MMR to violations of “what” predictions in a contextually specific fashion, such that more local (interval-based) “when” predictions modulated responses to deviant ID elements, while more global (beat-based) “when” predictions modulated responses to deviant BD elements, indicating a congruence effect in the processing of “what” and “when” predictions across different predictive contexts in the auditory system. While “what” and “when” predictions showed interactive effects for both contexts, BD-deviants in beat-based sequences and ID-deviants in interval-based sequences were associated with similar spatiotemporal patterns of EEG evoked activity modulations, and linked in the DCM analysis to a shared widespread connectivity increase at relatively late stages of cortical processing (between the STG and the fronto-parietal network). Interestingly, our findings contrast with related studies that have implicated separable neural mechanisms in the processing of beat-based and pattern/mnemonic-based “when” predictions (Breska and Deouell, 2017; Bouwer et al., 2020, 2023). Our results instead suggest that the interactions of “what” and “when” predictions, while contextually specific, are mediated by a shared and distributed cortical network independent of the type of “when” predictions.

Deviant responses to “what” prediction violations within melodic sequences and tone contours are well documented, having been used to explore a variety of phenomena in the auditory system (see Yu et al., 2015 for a partial review). Deviant tones within familiar musical scales have been found to elicit higher MMR amplitudes compared to those of unfamiliar scales are tones presented without a scale structure (Brattico et al., 2001), as well as higher deviant responses to out-of-scale notes in unfamiliar melodies (Brattico et al., 2006). Deviant responses to manipulated musical characteristics within melodic sequences (e.g., timing, pitch, transposition, melodic contour) have similarly been demonstrated in musician and non-musician groups (Vuust et al., 2011; Tervaniemi et al., 2014). In the predictive coding framework, such evoked responses can be understood in the context of prediction error, wherein bottom-up error signaling triggers the adjustment of higher-level models of the stimulus train formed as a result of perceptual learning during repeated stimulus presentation (Garrido et al., 2009a,b). Such hierarchical relationships have been quantified using DCM (Auksztulewicz and Friston, 2016), and are consistent with our analysis of the evoked responses observed herein. The resultant model shows increased connectivity throughout the network, consistent with increased error signaling (ascending connections), predictive template updates (descending connections), and gain connectivity evident in a decrease in gain following predictions errors. Our source reconstruction was equally consistent with existing literature revealing bilateral activity in the primary auditory cortex (A1) and higher-order auditory regions in the superior temporal gyrus (STG), as well as the right inferior frontal gyrus (IFG) (Garrido et al., 2008; Giroud et al., 2020).

In the context of “when” predictions, the results of our frequency domain analysis show that the EEG spectrum largely follows that of the stimulus spectrum, with several notable differences likely reflecting perceptual processing of sequences (Henry et al., 2017). While the EEG-based ITPC response at element-rate was stronger near central electrodes, consistent with previous EEG studies (Ding et al., 2017), the pair-rate effect was predominantly present in the right hemisphere, suggesting that the contextual structure of non-linguistic sequences can be entrained by parallel neural activity in different regions at distinct time scales – consistent with existing research (Giroud et al., 2020). Interestingly, the ITPC differences between conditions (interval-based vs. beat-based) emerged during the experiment in pair-rate peaks, but not in element-rate peaks, suggesting that rapid learning could modulate neural entrainment to auditory sequences with different regularities at the pair-rate level. Similarly, a previous study (Moser et al., 2021) found significant differences in non-linguistic triplet-rate ITPC peaks between structured and random conditions, occurring during early exposure. This ITPC difference suggests a fine shift in sequence encoding, with different regularities from single elements to integrated chunks. Notably, we also found correlations between the ITPC difference conditions and the congruence effect of ERP amplitude, indicating a set of neural correlates similarly sensitive to neural entrainment and the interaction of “what”/“when” predictions.

In addition to their dissociable main effects on neural activity, “what” and “when” predictions modulated element-evoked response amplitude interactively and in a contextually specific manner, such that interval-based “when” predictions amplified MMRs to “what” prediction violations falling on the interval-final time points (ID elements), while beat-based “when” predictions amplified MMRs to “what” prediction violations falling on the beat (BD deviants). These findings extend the result of previous studies, which showed that “when” predictions modulate MMR amplitude (Yabe et al., 1997; Takegata and Morotomi, 1999; Todd et al., 2018; Lumaca et al., 2019; Jalewa et al., 2020), by showing that these modulatory effects are congruent with respect to the expected time points, independent of the type of “when” predictions (interval-based vs. beat-based). Dynamic causal modeling of our ERP data showed partially dissociable connectivity patterns between the main effect of “what” predictions (i.e., all deviants vs. all standards), which increased recurrent connectivity throughout the network (Garrido et al., 2008; Auksztulewicz and Friston, 2015; Fitzgerald et al., 2021), and “what”/"when” interactive effects, which had a more nuanced pattern of effects on neural activity. Specifically, congruent “what” and “when” predictions decreased recurrent connectivity at lower parts of the network (between A1 and the STG), while at the same time increasing recurrent connectivity at higher parts of the network (between STG and the fronto-parietal regions). Previous DCM work has shown similar dissociations between processing deviants based on violations of relatively simple predictions vs. complex contextual information, indicating the higher-order regions as sensitive to complex prediction violations (Fitzgerald et al., 2021). Additionally, in the current results, the main effect of “what” predictions and the contextually specific integration of “what” and “when” predictions had opposing effects on the neural gain estimates for the left SPL region, which displayed decreased self-inhibition (increased gain) following deviant processing but increased self-inhibition (decreased gain) following prediction integration. These results mirror our source reconstruction, wherein deviants presented in congruent temporal conditions were associated with decreased left parietal activity, and imply the left parietal cortex – recently shown to mediate the integration of “what” and “when” information in speech processing (Orpella et al., 2020) – in the more general process of integrating “what” and “when” predictions also for non-speech stimuli. While this inhibitory effect of prediction integration observed at the source level may seem at odds with the results at the sensor level, where deviant-evoked ERPs in congruent conditions had an increased amplitude, it should be noted that these ERP components had a negative polarity (Figure 2C). Negative EEG deflections can arise due to superficial excitatory inputs or deep inhibitory inputs (Kirschstein and Köhling, 2009), raising the possibility that sensor-level and source-level results may be jointly explained by inhibition in deep layers in the parietal cortex. It is also worth noting that while “when” predictions did not elicit a significant main effect on ERP amplitude, it is possible this finding may have resulted from design constraints, as all conditions contained only “what” (repetition detection) tasks, suggestive of previous studies on the role of attention in parallel temporal and mnemonic predictive processing (Lakatos et al., 2013; Wollman and Morillon, 2018).

While in the EEG literature on MMN and other mismatch responses, different ERP components such as N100, P200 and N200 have traditionally been interpreted in functional terms as signatures of dissociable mechanisms (for reviews, see, e.g., Joos et al., 2014; Fitzgerald and Todd, 2020), our study uses computational modeling to explain MMR over the entire ERP time course in terms of underlying connectivity and gain effects (e.g., Garrido et al., 2008; Boly et al., 2011; Auksztulewicz and Friston, 2015; Todd et al., 2023). As such, the relative difference in latencies between the interactive effect of “what” and “when” congruence (130–180 ms relative to tone onset) and the main effect of “what” predictions (173–223 ms) can be directly interpreted by referring to DCM results, as likely originating from different patterns of connectivity in the network encompassing auditory and frontoparietal sources. The congruency effect was linked to a connectivity pattern limited to feedforward and intrinsic (gain) connections, supporting the idea that prediction error signaling increases when processing in temporally congruent conditions. Conversely, the main effect of “what” predictions was linked to both feedforward and feedback connections, consistent with previous studies showing a preferential engagement of feedback connectivity for later ERP latencies (Garrido et al., 2007). Therefore, the relatively later latency of the the ERP difference of deviants vs. standard may be primarily linked to the added contribution of top-down connections throughout the network.

Previous studies have shown that the processing of musical information requires predictive mechanisms for timing of content of auditory events, and that such predictions can have modulatory effects at different cortical levels when presented within the framework of melodic expectation (Royal et al., 2016; Di Liberto et al., 2020). Musical stimuli presents us with an intriguing opportunity to investigate predictive coding mechanisms, as the statistical regularities within musical frameworks are well defined and intrinsically learned. In particular, such structures allow us to disassociate “what” and “when” predictions while keeping other elements of a stimulus stream intact across manipulations and trials. Because the presence of musical syntax violations require knowledge acquired through long-term repeated exposure to music, long-term memory recall is also involved in establishing those regularities. Recent studies have probed beat-based and memory-based predictive processing through a variety of paradigms. Previous work has demonstrated that the rhythmic and mnemonic predictions cannot be fully accounted for with general entrainment models (Breska and Deouell, 2017) and that the processing of beat-based and memory-based auditory predictions may rely on different underlying mechanisms (Bouwer et al., 2020, 2023). Of note, the latter studies used a single percussive sound as their sole stimuli in designing rhythmic and mnemonic blocks, whereas our design taps into both rhythmic and semantic predictions within the context of western musical scales, suggesting that the hierarchical structure and complexity of stimulus streams may play a role in mediating the type of predictive mechanisms that occur in the brain. The role of memory in syntactical prediction violation is indeed an avenue ripe for further investigation, and future studies may wish to extend our paradigm to further probe the observed late-series ITPC pair-rate differences in that context. Furthermore, as “what” and “when” predictions are also ubiquitous in other stimulus domains – most prominently in speech perception (Emmendorfer et al., 2020) – future research should test whether similar contextual specificity of “what” and “when” predictions as observed here also govern speech processing.

### Significance statement

Predictions of stimulus features, present in different statistically-regular contexts in the environment, are crucial to forming adaptive behavior. However, it is unknown if the brain integrates predictions selectively according to such contextual differences. By recording human electroencephalography during experimental manipulations of time-based and content-based predictions, we found that those predictions interactively modulated neural activity in a contextually congruent manner, such that interval-based (vs. beat-based) temporal predictions modulated content-based predictions errors of interval-defining elements (vs. beat-defining elements). These modulations were shared between contextual levels in terms of the spatiotemporal distribution of neural activity. This suggests that the brain integrates different predictions with a high degree of contextual specificity, but in a shared and distributed cortical network.

## Data availability statement

The data set underlying the findings described and used to reach the conclusions of the manuscript is uploaded to the following Figshare repository: DOI 10.6084/m9.figshare.23959278.

## Ethics statement

The studies involving humans were approved by City University of Hong Kong Human Subjects Ethics Sub-Committee. The studies were conducted in accordance with the local legislation and institutional requirements. The participants provided their written informed consent to participate in this study.

## Author contributions

DC, JS, and RA designed research. WL and FP performed research. RA, DC, and DL analyzed data. DC, JS, and RA wrote the paper. All authors contributed to the article and approved the submitted version.

## Funding

This work has been supported by the European Commission’s Marie Skłodowska-Curie Global Fellowship (750459 to RA) and a grant from the European Commission/Hong Kong Research Grants Council Joint Research Scheme (9051402 to RA and JS).

## Conflict of interest

The authors declare that the research was conducted in the absence of any commercial or financial relationships that could be construed as a potential conflict of interest.

## Publisher’s note

All claims expressed in this article are solely those of the authors and do not necessarily represent those of their affiliated organizations, or those of the publisher, the editors and the reviewers. Any product that may be evaluated in this article, or claim that may be made by its manufacturer, is not guaranteed or endorsed by the publisher.
